# Hybrid Raman-erbium random fiber laser with a half open cavity assisted by artificially controlled backscattering fiber reflectors

**DOI:** 10.1038/s41598-021-88748-w

**Published:** 2021-04-28

**Authors:** R. A. Perez-Herrera, P. Roldan-Varona, M. Galarza, S. Sañudo-Lasagabaster, L. Rodriguez-Cobo, J. M. Lopez-Higuera, M. Lopez-Amo

**Affiliations:** 1 Department of Electrical Electronic and Communication Engineering, Public University of Navarra, 31006 Pamplona, Spain; 2 Institute of Smart Cities (ISC), Public University of Navarra, 31006 Pamplona, Spain; 3 Photonics Engineering Group, University of Cantabria, 39005 Santander, Spain; 4 CIBER-Bbn, Instituto de Salud Carlos III, 28029 Madrid, Spain; 5 Instituto de Investigacion Sanitaria Valdecilla (IDIVAL), 39005 Santander, Spain

**Keywords:** Optics and photonics, Lasers, LEDs and light sources, Fibre lasers

## Abstract

A hybrid Raman-erbium random fiber laser with a half-open cavity assisted by chirped artificially controlled backscattering fiber reflectors is presented. A combination of a 2.4 km-long dispersion compensating fiber with two highly erbium-doped fiber pieces of 5 m length were used as gain media. A single random laser emission line centered at 1553.8 nm with an optical signal to noise ratio of 47 dB were obtained when pumped at 37.5 dBm. A full width at half maximum of 1 nm and a 100% confidence level output power instability as low as 0.08 dB were measured. The utilization of the new laser cavity as a temperature and strain sensor is also experimentally studied.

## Introduction

In recent years, random fiber lasers (RFLs) have thoroughly shown valuable applications in many practical engineering requests such as remote sensing, communications, optical astronomy and biomedical images among others^1^. One of their main characteristics is that they do not have well-defined mirrors but distributed reflectors instead, their feedback depending on the scattering along the fiber^2^. Such optical fiber distributed reflectors using Rayleigh backscattering provide interesting properties in lasers. They suppress multiple longitudinal modes into the cavity, generating a modeless behavior^3^. They also lead to low spatial and/or temporal coherence^4^. This type of random lasers also show a high output power stability^1^, high efficiency^5^ and simplicity of fabrication^6^.

RFLs strongly depend on the scattering properties of the medium^7–12^, the pump profile^13^ and the geometry of the active zone^14^. Rayleigh scattering (RS) of the light is a consequence of random density fluctuations which cause random refractive index variations in the medium^15^. This scattering offers a feedback mechanism that generates a distributed reflector in random lasers^16^. Most of the initially presented configurations for random fiber lasers needed several tens of kilometers of single mode fiber (SMF) as gain medium^17^ due to its low Rayleigh backscattering coefficient, around 10^−7^ m^−1^^18^. However, by using other type of fibers such as dispersion compensating fibers (DCFs), with Rayleigh backscattering coefficients up to 7 times higher than SMF, and a higher Raman gain, cavity length can be reduced to several kilometers ^18^.

Hybrid erbium and Raman gain have also been used to provide amplification for the weak RS feedback amplification to enhance this feedback, and therefore generate single or multiple wavelength RFLs^15,19,20^. Random feedback from RS can also be enhanced by drilling the optical fiber in order to generate distributed or quasi-distributed reflectors. These drilled fibers, termed as artificially controlled backscattering fiber reflectors (ACBFRs), are fabricated by using a femtosecond laser with ultrashort pulses width and very high instantaneous power. These pulses induce a nonlinear multi-photon absorption in the material causing a modulation of the refractive index along the optical fiber^21,22^. Moreover, both reflectivity and longitudinal position, as well as the distribution of these scattering points can be controlled.

In this work, a hybrid Raman-erbium random fiber laser (RFL) with a half-open cavity, based on chirped ACBFRs is presented. The relationships between the pump power level and the full width at half maximum (FWHM) with the observed output power instability are experimentally demonstrated. Laser´s cavity properties in terms of spectral characterization, output power and wavelength stability are presented and compared with the results obtained with fiber Bragg gratings (FBGs). The sensing response of the laser as a function of temperature and strain variations is also experimentally demonstrated.

## Working principle: simulation and inscription process

The reflector based on chirped ACBFRs were manufactured by using a commercial femtosecond fiber laser chirped pulse amplifier (FLCPA) with a 1030 nm operating wavelength and 370 fs pulse duration. These pulses were tightly focused with a NA = 0.42 Mitutoyo objective lens onto the fiber to induce refractive index variations in the core of around 10^–3^. The fiber is located on a nano-resolution air-bearing XYZ stage from Aerotech for precise displacement.

The pattern of the required defects along the fiber was designed using the software *Fimmwave* from Photon Design^23^, a vectorial-mode solver for 2D + Z waveguide structures that can model propagation in 2D and 3D structures. The reflectors had to meet two specifications: (1) they should be wide enough to allow this laser structure to have a modeless behavior, and (2) they should be centered at around 1550 nm matching the gain zone. A sequence of defects in the fiber 3 µm wide and 1 µm long separated an increasing distance from 1.08 to 1.13 µm best fitted these requirements. Due to technical limitations the ACBFR were 2.5 mm long.

**Figure 1:**
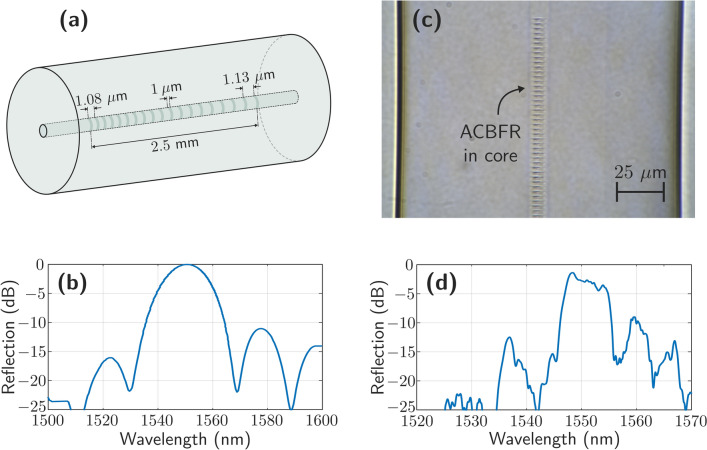
(**a**) Schematic of the calculated chirped ACBFRs. (**b**) Simulated output spectrum of the chirped ACBFR. (**c**) Microscope image of the manufactured ACBFR. (**d**) Output spectrum of the manufactured chirped ACBFR measured by an optical spectrum analyzer.

A pulse energy of 4.5 µJ (before the slit) and a 100 Hz pulse repetition rate (PRR) were used for the drilling. The chirped reflector presents a period between consecutive pulses that evolves from ${\Lambda }_{min} = 2.1423$ µm to ${\Lambda }_{max} = 2.1533$ µm, which generates the 4th harmonic in the C-band. It must be noted that the slit beam shaping technique has been used, through which it is possible to modify the focal volume, and thus achieve greater control over the dimensions of each refractive index change^24^. This aspect can be seen in the inscribed optical structure shown in Fig. 1c.

Figure 1a shows the schematic of the designed reflector and Fig. 1b depicts its calculated reflected output spectrum. Figure 1c presents a microscope image of the manufactured ACBFR and Fig. 1d illustrates its spectrum measured with an optical spectrum analyzer (OSA). A maximum power reflection value of − 1.37 dB centered at around 1548.4 nm and a FWHM of 7.45 nm were measured. The two chirped ACBFRs used in this work showed very similar spectra. Figure 1c shows a microscope image of one of these manufactured ACBFRs.

## Experimental setup

**Figure 2:**
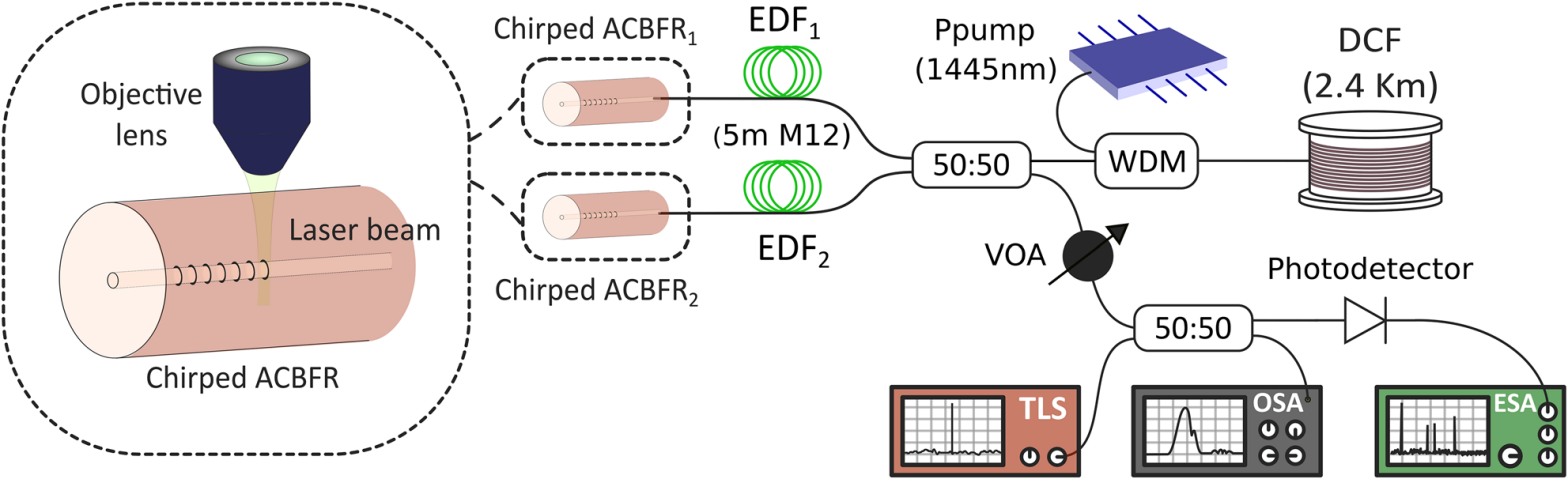
Experimental setup of the experimental half-open cavity RFL based on two chirped artificially controlled backscattering fiber reflectors (ACBFRs).

The schematic diagram of the experimental setup of the half-open linear cavity RFL assisted by a chirped ACFBR is shown in Fig. 2. A 1445/1550 nm wavelength division multiplexer (WDM) injects the Raman pump power centered at 1445 nm into the RFL. At one of its ends, a 2.4 km length spool of DCF, acting as a distributed reflector and amplifying the randomly backscattered light was located. As aforementioned, the DCF provides additional Rayleigh scattering feedback for the RFL^2^, inducing a significant reduction of the typical gain medium length^25^. At the opposite end of the RFL, a 3 dB optical coupler divides the optical signal reflected by the DCF into two different branches, using a single gain medium per wavelength^26^. Each one of these branches includes 5 m of highly erbium-doped fiber (EDF) followed by a fabricated chirped artificially controlled backscattering fiber used as wavelength selected mirror. The used EDF was the M12 (980/125) from Fibercore Inc., with a theoretical peak core absorption range from 16 to 20 dB/m at 1531 nm.

The reflected signals from these two branches pass through a variable optical attenuator (VOA) in order to minimize the risk of damage of the measurement devices due to high power levels. Simultaneous measurements in the optical and electrical domain were carried out by dividing this signal with another 3 dB optical coupler. One output branch was connected to an optical spectrum analyzer (OSA) with a resolution of 0.1 nm and a sensitivity of − 75 dB.

In order to experimentally verify the modeless behavior of the RFL, the other output branch was connected to a photodetector in combination with an electrical spectrum analyzer (ESA) to perform measurements in the electrical frequency domain. The RFL output, reflected from the chirped ACBFRs, were mixed with the signal of a tunable laser source (TLS, Agilent 8164B), though a 3 dB coupler to perform a heterodyne detection.

Free ends of chirped ACBFRs and DCF were immersed in refractive-index-matching gel to avoid undesired reflections. All the experimental measurements were carried out at room temperature, and no vibration isolation or temperature compensation techniques were employed.

## Results and discussion

### Spectral characterization

**Figure 3:**
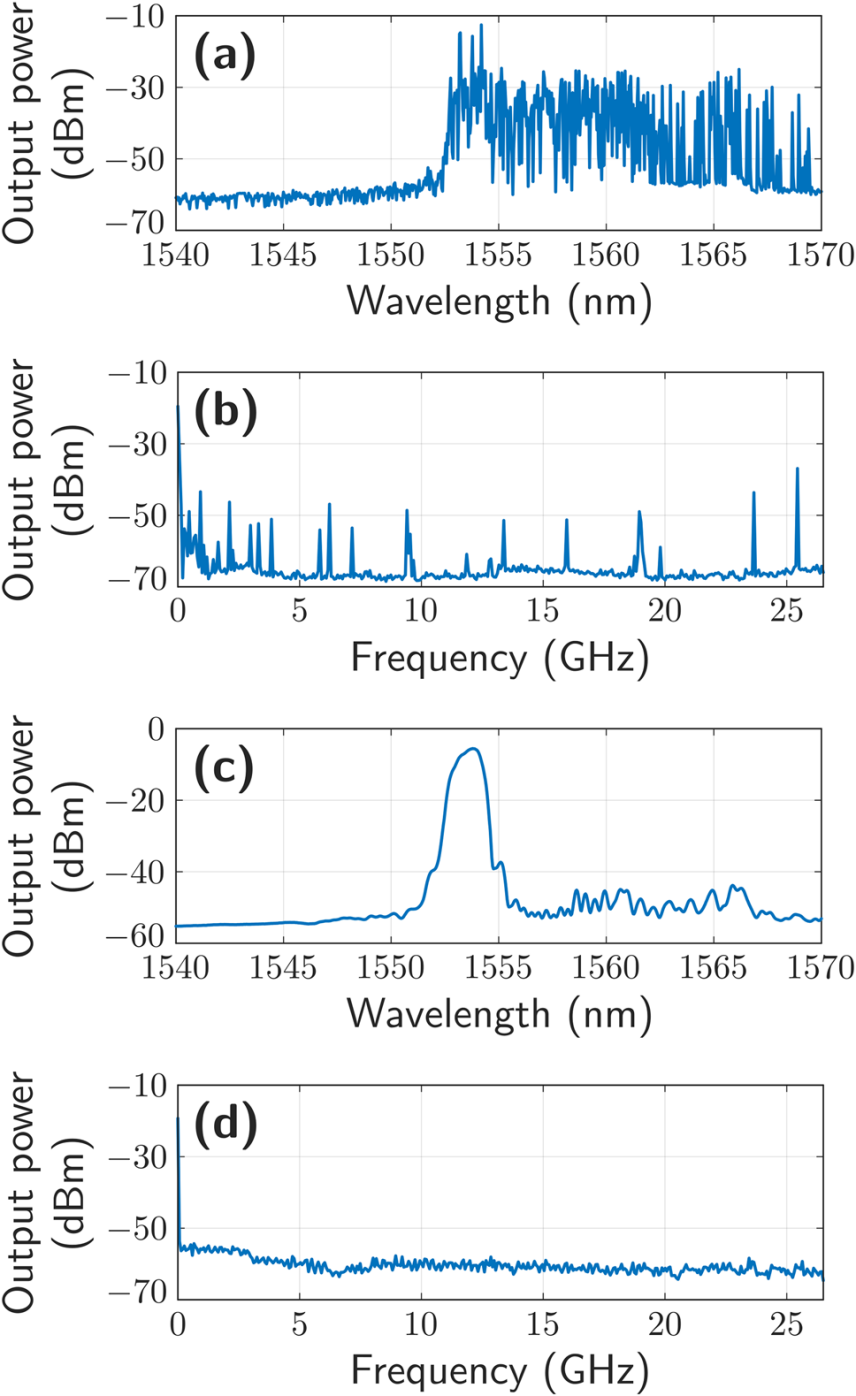
Output spectra of the RFL measured in the optical domain when pumped at 32.5 dBm (**a**) and 37.5 dBm (**c**). Radio-frequency spectra of the beat of the RLF with the TLS when pumped at 32.5 dBm (**b**) and 37.5 dBm (**d**).

To evaluate the output spectra of the laser, the half-open cavity RFL was pumped with powers ranging from 32.5 up to 37.5 dBm. Figure 3a, b show the measured optical and electrical spectra, respectively, when pumped at 32.5 dBm. Both cases illustrate that modeless behavior was not obtained for such a low pump power level. On the other hand, when Raman pump power is increased up to 37.5 dBm, random laser behavior is experimentally ratified as Fig. 3c, d illustrate.

In all cases, the backscattered light forms a multitude of resonant modes with random frequencies^17^. Nevertheless, the reflection from the chirped ABCFs was dominant in the half-open cavity and only the resonant modes of these reflectors reached their threshold (when the combination of the EDFA and Raman gain overcame the cavity loss).

The spectrum shape presented in Fig. 3a shows a great number of wavelength emission lines from 1552 up to 1570 nm with output power levels of around -30 dBm. However, when pump power was increased to 37.5 dBm, a random laser emission line centered at 1553.8 nm was measured, as it can be in Fig. 3c. The output power level obtained from this RFL was − 6.5 dBm with a full width at half maximum (FHWM) of 1 nm, and an optical signal to noise ratio (OSNR) of 47 dB.

Figure 3b, d illustrate the frequency spectra corresponding to the frequency domain conversion, when a photodetector in combination with an ESA was used to perform the measurements. There is no indication of longitudinal mode beating in Fig. 3d, when pumped at 37.5 dBm. Nevertheless, Fig. 3b clearly shows the appearance of longitudinal mode beating, which varies as a function of the pump power level. These results support the modeless theory of random fiber lasers reported in^27^.

**Figure 4:**
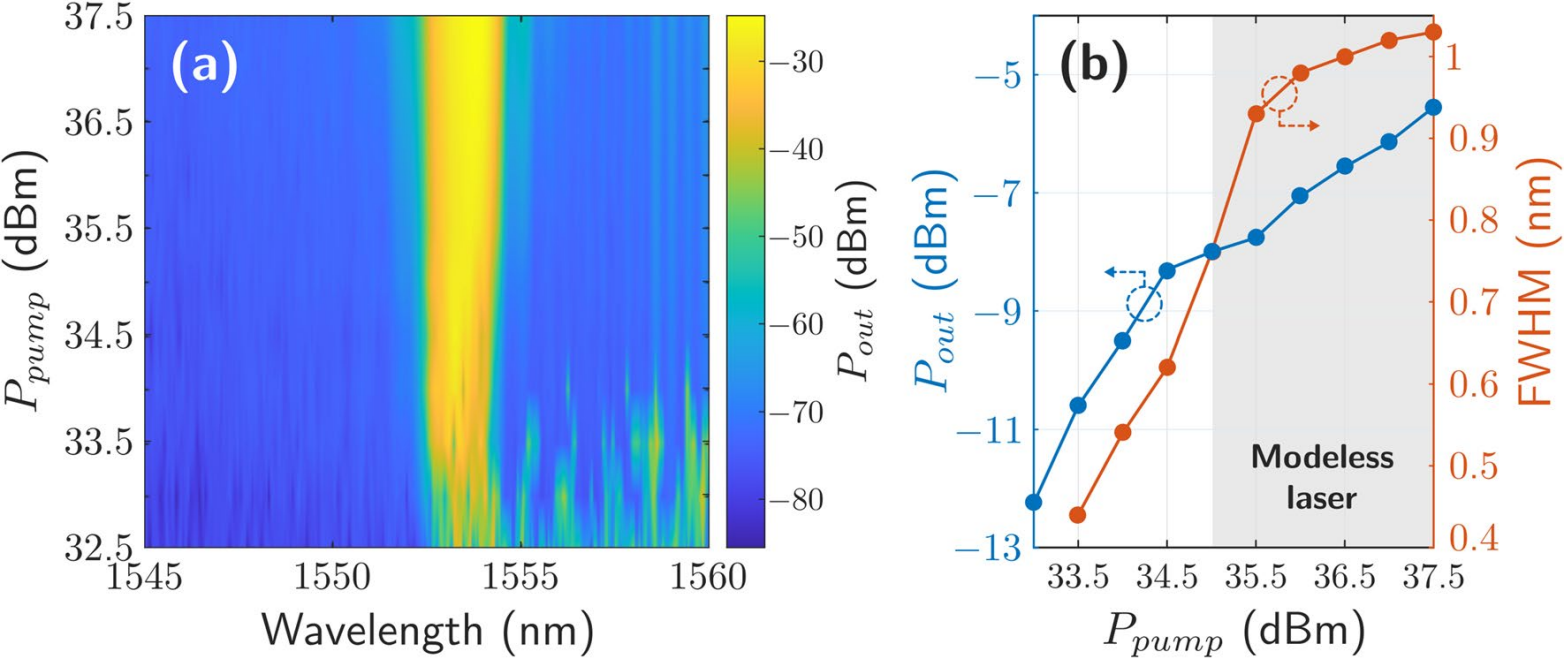
(**a**) Relationship between the pump power and output power levels as a function of wavelength (MATLAB R2020b, contourf command. https://es.mathworks.com/products/matlab.html). (**b**) Relationship between the output power and FHWM values as a function of pump power.

In order to characterize the dependency of the output power level, FWHM and modeless laser behavior with the pump power a sweep from 32.5 up to 37.5 dBm in 0.5 dB steps was carried out. Figure 4a displays the experimental results of the measured output power level as a function of the inserted pump power. The central wavelength emission of the RFL was maintained around 1553.8 nm along the measurement range. However, this is not the case for the FHWM since it widens for higher pump power levels. Figure 4b shows the output power and the FHWM as a function of pump power level. It can be clearly seen that the obtained RFL does not reach a modeless laser behavior until the pump power does not reach 35 dBm, which corresponds to a FWHM value of 0.76 nm.

### Output power stability

**Figure 5:**
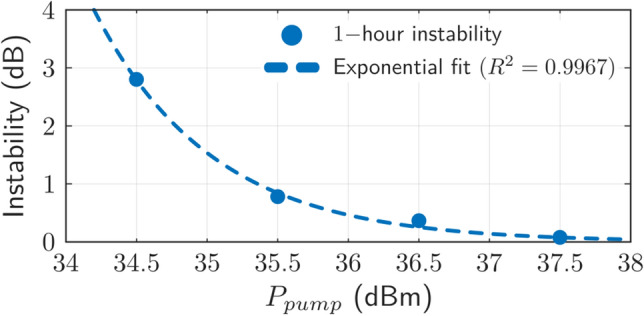
Output power instability as a function of Raman pump power, measured over 1 h with a confidence level of 100%.

The output power level and wavelength stability of a fiber laser are key parameters to be assessed, since these output power variations have high influence on the precision of sensor systems. High intensity stability guarantees high resolution operation of the intensity sensor^28^. Figure 5 shows the output power instability of the half-open RFL as a function of the inserted pump power. The measured data was stored each 10 s for one hour considering a confidence level (CL) of 100%. It was observed that the peak power averaged at room temperature presented a variation as low as 0.08 dB when a pump power level of 37.5 dBm was inserted. These results present a remarkable improvement compared to previous and recent works^29^. As Fig. 5 shows, an exponential relationship between pump power and output power instability was experimentally evaluated, with an error of R^2^ = 0.9967. Therefore, as the output spectrum becomes wider, that is, the FWHM increases, the output power stability increases considerably.

**Figure 6:**
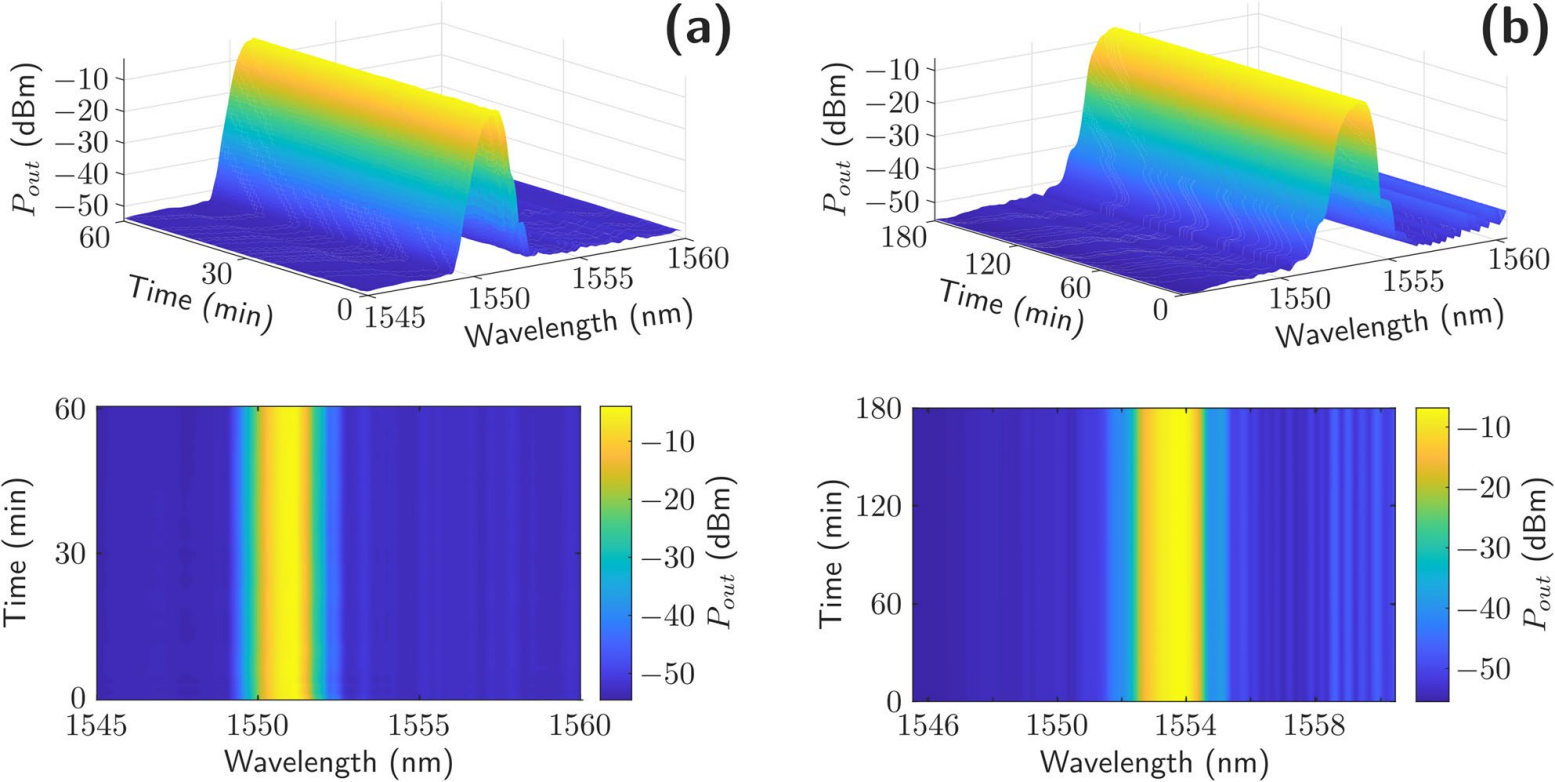
(**a**) Output power stability when pumped at 37.5 dBm by using FBGs (**a**) or chirped ACBFRs (**b**) as reflectors as a function of time. (MATLAB R2020b, surf and contourf commands for the upper and lower figures respectively. https://es.mathworks.com/products/matlab.html).

To quantify the improvement when using chirped ACFBRs, the measurements were repeated using two fiber Bragg gratings (FBGs) centered at 1551 nm as reflectors. Figure 6a shows the measured output power stability when pumped at 37.5 dBm by using FBGs as reflectors to be compared to the results provided by the chirped ACBFRs, presented in Fig. 6b. In both cases, measured data was stored each 10 s over one hour and a confidence level (CL) of 100% was considered. Even though the optical spectrum shape obtained with the FBGs does not present lateral lobes, its stability is notably worse. Moreover, both measured emission lines, centered at 1551 nm (Fig. 6a) and 1553.8 nm (Fig. 6b) deal with the same gain shape. The output power level presented a variation of 0.53 dB when using FBGs at room temperature, but only 0.08 dB when using ACFBRs. Therefore, the use of ACFBRs implies a remarkable increase of the output power level stability under identical circumstances: around six time higher.

### Sensing response

**Figure 7:**
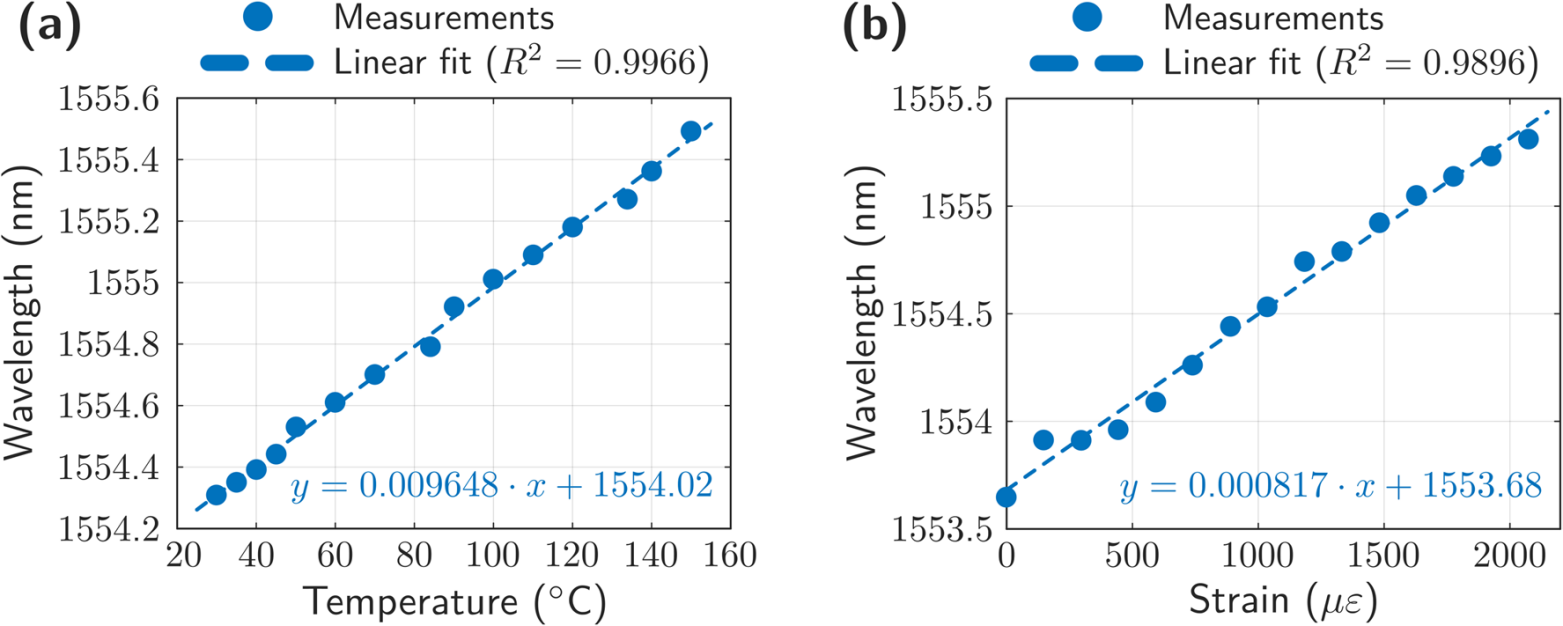
Wavelength shift of the half-open RFL assisted by chirped ACBFRs as a result of (**a**) temperature and (**b**) strain variations.

The utilization of this half-open RFL in sensing applications was also analyzed. We tested the chirped ACBFRs as temperature and strain sensor heads. The response of the reflectors to the temperature was checked by heating them in a climatic chamber from 25 to 150 °C. As it can be seen in Fig. 7a, the center wavelength shift for the laser presents a clear linear behavior (the mean square error is 0.9966, very close to 1) and a temperature sensitivity of 9.6 pm/°C.

Next, these sensor heads based on chirped ACBFs, were placed in a high precision single-axis motorized stage (MS) in order to evaluate the wavelength shift produced by their mechanical stain. The sensor head characterization consisted of 28 steps of about 790 µɛ. As Fig. 7b illustrates, the strain response of these reflectors, when axial strain from 0 to 2100 µε was applied, also presented a linear behavior with a strain sensitivity of 0.8 pm/µε and an error of R^2^ = 0.9896.

Typically, FBG-based sensing systems offer a temperature sensitivity of 11 pm/°C and a strain sensitivity of 1.2 pm/µε^30,31^. These measurement sensitivities have been enough to establish this kind of sensors as a flagship in sensing along two decades or more. Here, both temperature and strain sensitivity measured values are not as good as the FBG-based ones. However, the measured values together with the achieved stability both in terms of output power and wavelength allows this RFL system assisted by chirped ACBFRs to be used as a suitable option for a number of sensing applications.

## Conclusions

In this work, a new hybrid Raman-erbium random fiber laser with a half-open cavity, assisted by chirped artificially controlled backscattering fiber reflectors has been presented. A combination of a 2.4 km-long dispersion compensating fiber with two highly erbium-doped fiber segments of 5 m were used as a gain media. By using these chirped ACBFRs, a single random laser emission line centered at 1553.8 nm and an optical signal to noise ratio of 47 dB have been measured when pumped at 37.5 dBm. A full width at half maximum of 1 nm and an output power instability as low as 0.08 dB, with a confidence level of 100% have been measured. Its sensor response as a function of temperature and strain variations has been also experimentally demonstrated.
